# Functional Interaction between Human Papillomavirus Type 16 E6 and E7 Oncoproteins and Cigarette Smoke Components in Lung Epithelial Cells

**DOI:** 10.1371/journal.pone.0038178

**Published:** 2012-05-25

**Authors:** Juan Pablo Muñoz, Carolina González, Bárbara Parra, Alejandro H. Corvalán, Maria Lina Tornesello, Yoshito Eizuru, Francisco Aguayo

**Affiliations:** 1 Virology Program, Instituto de Ciencias Biomédicas (ICBM), Faculty of Medicine, Universidad de Chile, Santiago, Chile; 2 Department of Hematology-Oncology, School of Medicine, Pontificia Universidad Católica de Chile, Santiago, Chile; 3 Molecular Biology and Viral Oncology and AIDS Reference Centre, National Cancer Institute “Fond. Pascale”, Naples, Italy; 4 Division of Oncogenic and Persistent Viruses, Center for Chronic Viral Diseases, Kagoshima University Graduate School of Medical and Dental Sciences, Sakuragaoka, Kagoshima, Japan; National Jewish Health, United States of America

## Abstract

The smoking habit is the most important, but not a sufficient cause for lung cancer development. Several studies have reported the human papillomavirus type 16 (HPV16) presence and E6 and E7 transcripts expression in lung carcinoma cases from different geographical regions. The possible interaction between HPV infection and smoke carcinogens, however, remains unclear. In this study we address a potential cooperation between tobacco smoke and HPV16 E6 and E7 oncoproteins for alterations in proliferative and tumorigenic properties of lung epithelial cells. A549 (alveolar, tumoral) and BEAS-2B (bronchial, non-tumoral) cell lines were stably transfected with recombinant pLXSN vectors expressing HPV16 E6 and E7 oncoproteins and exposed to cigarette smoke condensate (CSC) at different concentrations. HPV16 E6 and E7 expression was associated with loss of p53 stability, telomerase (hTERT) and p16^INK4A^ overexpression in BEAS-2B cells as demonstrated by quantitative real-time polymerase chain reaction (qRT-PCR) and western blotting (WB). In A549 cells we observed downregulation of p53 but not a significant increase of hTERT transcripts. In addition, the HPV16 E6/E7 transfected cell lines showed an increased proliferation rate and anchorage-independent growth in a HPV16 E6 and E7 expression-dependent manner. Moreover, both HPV16 E6/E7 and mock transfected cells showed an increased proliferation rate and anchorage-independent growth in the presence of 0.1 and 10 µg/mL CSC. However, this increase was significantly greater in HPV16 E6/E7 transfected cells (p<0.001). Data were confirmed by FCSE proliferation assay. The results obtained in this study are suggestive of a functional interaction between tobacco smoke and HPV16 E6/E7 oncoproteins for malignant transformation and tumorigenesis of lung epithelial cells. More studies are warranted in order to dissect the molecular mechanisms involved in this cooperation.

## Introduction

Human Papilloma Virus (HPV) infection and persistence are the necessary conditions for cervix-uterine cancer development [1], [2]. In addition, HPV has been identified as an etiological agent in a subset of head and neck cancers [3] and has been detected in extragenital tumors such as those affecting the esophagus and lungs, among others [4]. The establishment of an etiological association between HPV and lung cancer has been difficult because of the high variability of HPV prevalence in lung tumors from one country to another [5]. Moreover, it is known that lung cancer development is strongly and directly related to tobacco smoking or cigarette smoke exposition. However, a very low percentage of heavy smokers finally develop lung cancer, suggesting that other co-factors are necessary [6]. It has been suggested that HPV has a role as an independent carcinogen when this virus is found in non-smoking subjects, as occurs in women of Taiwan who develop lung adenocarcinomas [7], [8]. However, other studies have reported a variable presence of HPV in smoking subjects who develop lung cancer [9], thus the role of HPV in these tumors remains to be elucidated. It is plausible that HPV in these cases might act as a co-carcinogen [10], however functional evidence for a potential cooperation between HPV and tobacco smoke for lung tumorigenesis is lacking.

So far, HPV16 has been the most frequent high-risk HPV genotype found in lung carcinomas around the world [5] with frequent E6/E7 oncogene expression [11]. High-risk HPV integration into the host genome [12] was previously reported to occur in lung cancer [13]. This event allows the overexpression of E6 and E7 oncoproteins due to the loss of E2 protein, a repressor of the p97 promoter in HPV16 [14]. The high-risk HPV E6 oncoprotein [15] is able to bind to the p53 protein, whose main function is sensing the integrity of DNA, inducing cell cycle arrest or apoptosis [16]. On the other hand, the high-risk HPV E7 oncoprotein [15] targets hypophosphorylated pRb for proteasome-dependent degradation through its association with the ubiquitin ligase complex [17]. This interaction allows the release of the transcription factor E2F, the expression of genes involved in G1/S transition, S phase entry and therefore DNA replication [18]. Furthermore, the specific loss of pRb leads to p16^INK4a^ up-regulation by a negative feedback mechanism [19]. In addition, high-risk E6 oncoprotein in collaboration with E7 are associated with alterations in the level and activity of telomerase (hTERT) [20].

The oncogenic role of tobacco smoking and cigarette smoke components (CSC) in lung cancer has been demonstrated in previous epidemiological and functional studies [21]. Even though the molecular mechanisms of tobacco smoke-associated carcinogenesis remain unclear, it has been established that benzopyrene, a polycyclic aromatic hydrocarbon (PAH) and potent carcinogen, activates the epidermal growth factor receptor (EGFR) and cell proliferation [22]. In addition, functional studies using organotypic cultures have demonstrated that benzopyrene, depending on its concentration, is able to increase the number of virions and genomes of HPV [23]. Using high concentrations of benzopyrene, virion synthesis is allowed. However, at low concentrations HPV genomes are amplified. The authors suggested that cyclin-dependent kinase 1 (CDK1), activated after incubation with benzopyrene may be involved in virion maturation; a mechanism used by diverse viruses in morphogenesis [24]. In addition, the same authors suggested that an increased CDK1 activity could be involved in viral persistence in the cells. Recently it has been reported that benzopyrene increases HPV E7 expression in a model of cervical raft cultures [25]. In addition to functional studies, epidemiological reports established that the smoking habit is a co-factor working synergistically for cervical carcinogenesis [26]. In fact, the International Agency for Research on Cancer (IARC, Lyon, France) established that tobacco smoke is a potential cervix-uterine carcinogen [27]. Thus the objective of this study was to analyze a potential cooperation between HPV16 E6/E7 oncoproteins and cigarette smoke components in lung epithelial cells exposed to both carcinogenic agents.

## Results

### Transfection of lung cell lines with pLXSNE6 and E7 constructs allows efficient expression of E6 and E7 transcripts and functional E6 and E7oncoproteins

**Figure 1:**
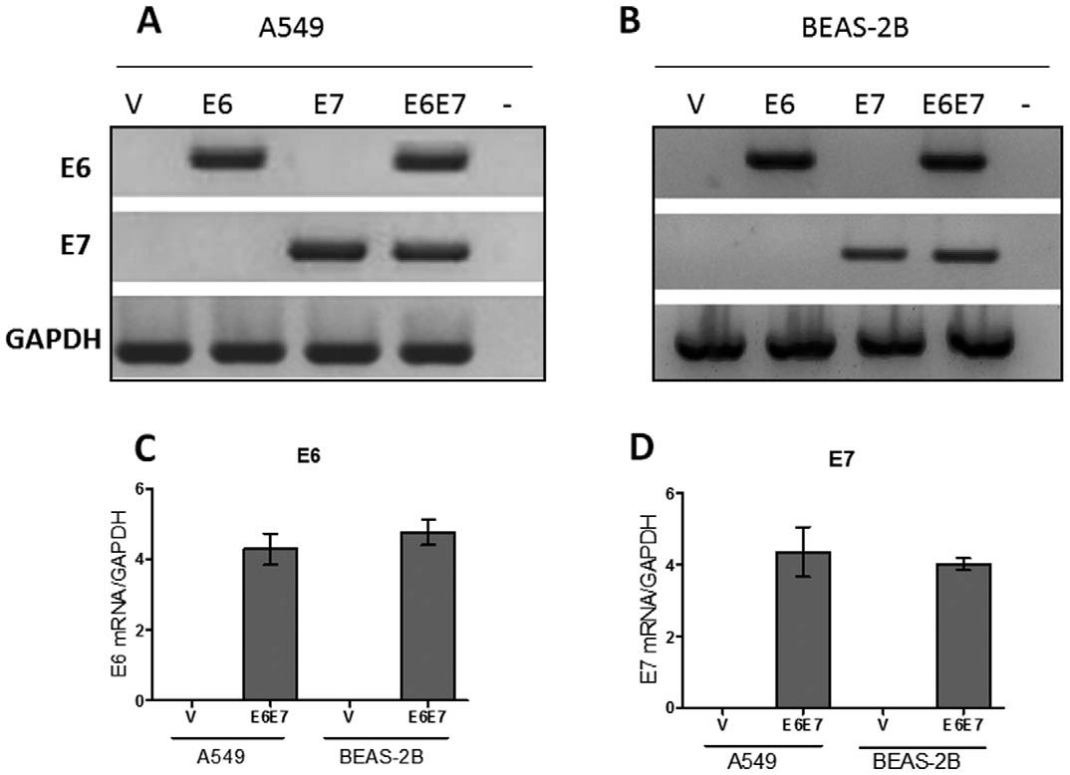
HPV-16 E6 and E7 transcripts are detected in lung cells stably transfected with pLXSNE6E7 constructs. **A**, **B**: Reverse-transcriptase PCR for HPV16 E6, E7 and GAPDH transcripts in A549 (**A**) and BEAS-2B (**B**) cells. **C**, **D**: qRT-PCR for HPV16 E6 (**C**) and E7 (**D**) mRNA in A549 and BEAS-2B cells transfected with pLXSNE6E7 constructs (mean ± SD).

**Figure 2:**
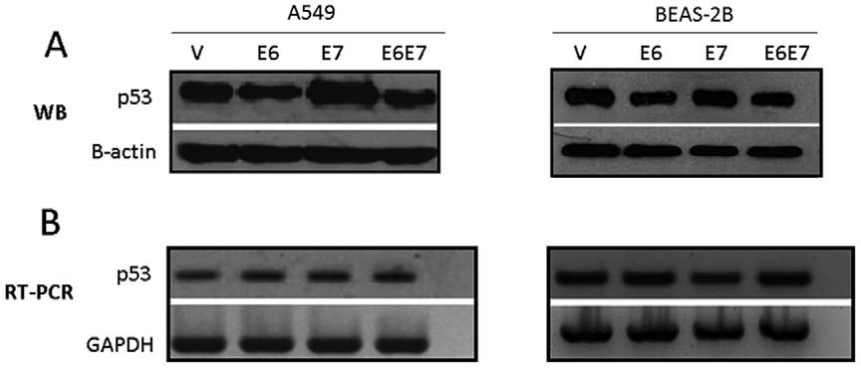
HPV16 E6 and E7 oncoproteins have functional activity in lung cells. **A**: Western blot for p53 protein in A549 and BEAS-2B transfected cells with the corresponding construct. **B**: Transcriptase-reverse PCR for p53 transcripts in A549 and BEAS-2B transfected cells.

Transfection of A549 and BEAS-2B lung cell lines with a pLXSN recombinant vector containing the HPV16 E6 and E7 open reading frames (ORF) allowed an efficient and stable expression of the corresponding transcripts (**Figs.** **1A and B**). In addition, both cell lines expressed similar levels of E6 and E7 transcripts that were not detected in cells transfected with the empty vector as shown using qRT-PCR (**Figs.** **1C and D**). The functional activity of the E6 oncoprotein was demonstrated in both cell lines by loss of p53 stability in HPV16 E6 and E6/E7 transfected cells as shown using WB (**Fig.** **2A**). As control, a semiquantitative RT-PCR assay with primers flanking the TP53 gene exon 1 showed the same level of p53 transcripts for empty vector, E6, E7 and E6/E7 in both A549 and BEAS-2B transfected cells (**Fig.** **2B**). In addition A549 cells transfected with the HPV16 E7 vector showed p53 up-regulation, demonstrating functional activity of this oncoprotein. Taken together, both HPV16 E6 and E7 oncoproteins are being expressed and are functional in A549 and BEAS-2B transfected cells.

### BEAS-2B cell lines transfected with pLXSNE6E7 constructs show increased hTERT and p16^INK4a^ expression

**Figure 3:**
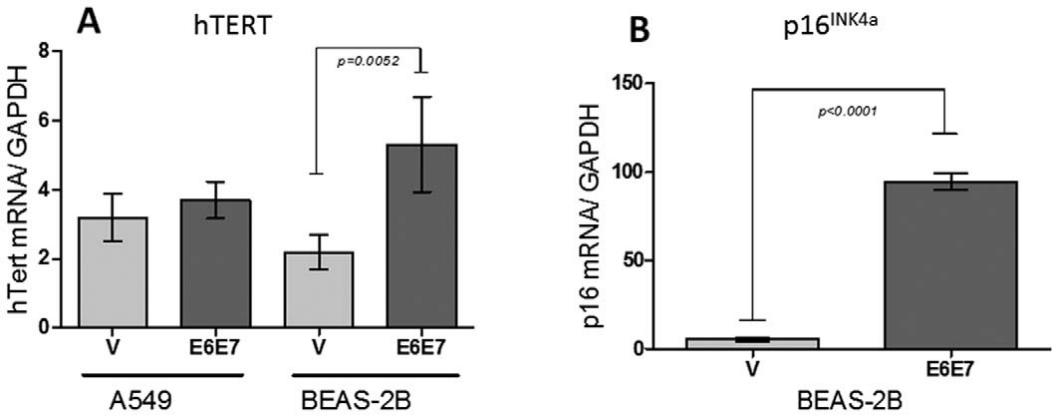
Telomerase (hTERT) and p16^INK4a^ transcripts are overexpressed in A549 and BEAS-2B cells transfected with pLXSNE6E7 constructs. **A**: hTERT mRNA detection is significantly increased in BEAS-2B cells transfected with HPV16 E6E7 constructs as detected using qRT-PCR (p=0.0052). **B**: p16^INK4a^ detection is significantly increased in BEAS-2B cells as detected using qRT-PCR (mean ± SD, p<0.0001, unpaired two-tailed Student's t-test).

In order to determine if HPV16 E6/E7 expression is able to alter the level of other genes in lung cells that are currently overexpressed in HPV-associated tumorigenesis, we evaluated changes in the expression level of telomerase and p16^INK4a^. As shown in **Fig.** **3**, even though the tumoral A549 cells constitutively express hTERT, the levels of these transcripts were not significantly increased after transfection with HPV16 E6 and E6/E7 oncogenes (p>0.05). However, BEAS-2B cells showed a statistically significant increase of telomerase transcripts as shown using qRT-PCR (**Fig.** **3A**). In addition, p16^INK4a^ showed a significant increase after transfection of BEAS-2B cells with E6 and E7 oncogenes (**Fig.** **3B**). Because A549 cells have a deletion into the region encoding for p16^INK4A^, this gene was not evaluated in these cells. Thus, the HPV16 E6 oncoprotein is able to induce hTERT expression in non-tumoral lung cells. Because of the increased hTERT expression in HPV16 pLXSE6E7 transfected cells, we worked with these constructs for subsequent experiments.

### Lung cells transfected with pLXSNE6E7 show increased proliferation rate

**Figure 4:**
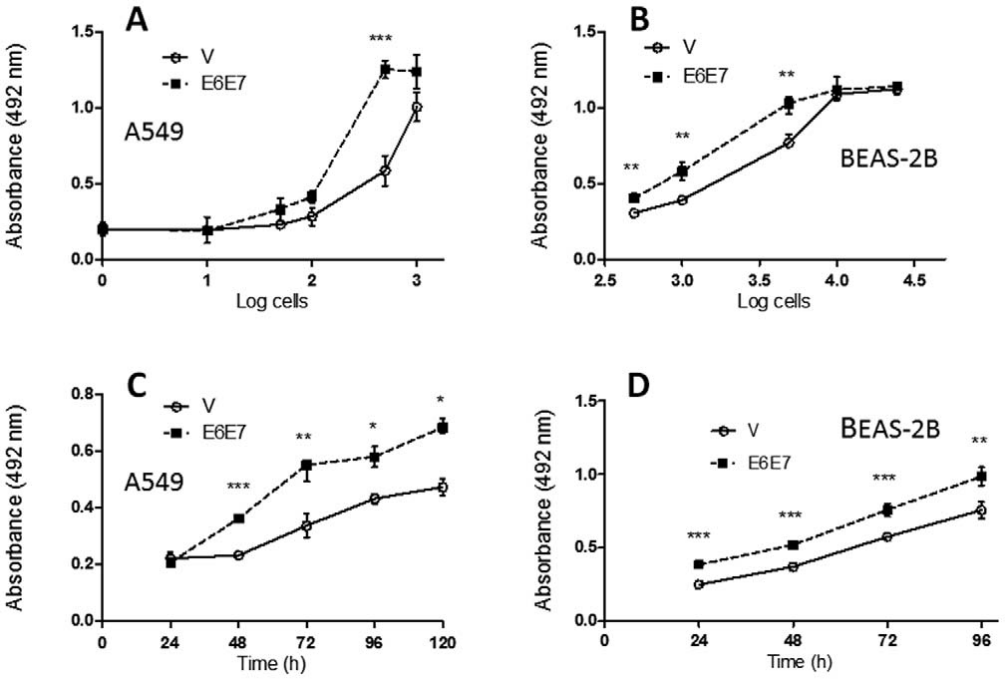
A549 and BEAS-2B lung cells transfected with pLXSNE6E7 constructs show an increased proliferation rate using MTS assays (mean ± **SE).** **A**, **B**: Viability of A549 (**A**) and BEAS-2B (**B**) lung cells transfected with the empty vector and pLXSNE6E7 constructs. **C**, **D**: Proliferation curves of A549 (**C**) and BEAS-2B (**D**) cells transfected with PLXSNE6E7 and empty vectors (mean ± SD, * p<0.05; ** p<0.01, *** p<0.001, unpaired two-tailed Student's t-test).

Using MTS assays we checked the viability and proliferation rate of HPV16 E6/E7 transfected cells and were compared with those transfected with the empty vector. We found that both A549 and BEAS-2B E6/E7 transfected cells showed a statistically significant increase of proliferation rate compared to the corresponding empty vector (**Fig.** **4**). The viability of A549 cells transfected with E6/E7 cultured during 72 h was increased in all the concentration range (**Fig.** **4A**). In addition, A549 cells showed an increased proliferation rate until 120 h of incubation (**Fig.** **4C**). Non-tumoral BEAS-2B cells showed an increase in viability and proliferative behavior when transfected with HPV16 E6/E7, even though this change was less evident compared to tumoral A549 cells (**Fig.** **4B and D**).

### Lung cells transfected with PLXSNE6E7 and acutely exposed to CSC increase significantly the proliferation rate

**Figure 5:**
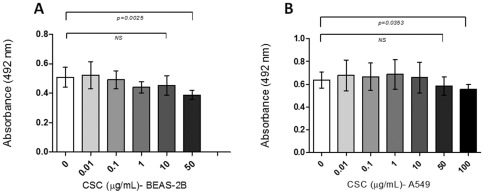
Viability of BEAS-2B (A) and A549 (B) lung cells transfected with pLXSN constructs and exposed to cigarette smoke condensate (range: 0–100 μg/mL) (mean ± SD, unpaired two-tailed Student's t-test). NS: Non-significant.

**Figure 6:**
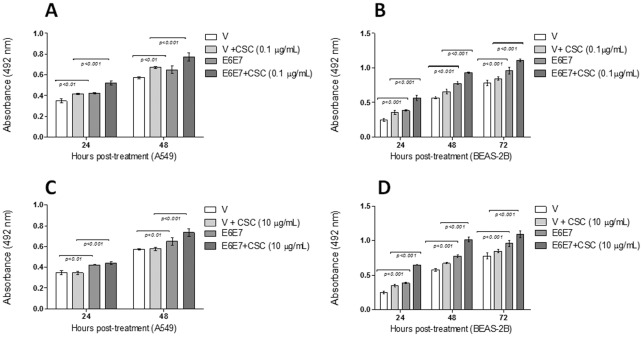
A549 (A, C) and BEAS-SB (B, D) cells transfected with pLXSNE6E7 constructs and exposed to 0.1 and 10 **µg/mL CSC show an increased viability and proliferation rate (mean** ± SD, * p<0.05; ** p<0.01, *** p<0.001, unpaired two-tailed Student's t-test).

**Figure 7:**
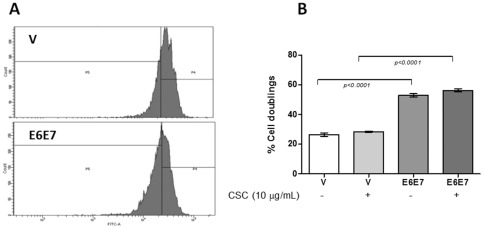
CFSE-labeled A549 cells transfected with empty vector and pLXSNE6E7 were exposed during 48 **h to 0.1** **µg/mL of CSC.** A representative histogram is shown (mean ± SD, unpaired two-tailed Student's t-test).

In order to determine the CSC concentration to be used in the transfected cells, we assayed the viability of A549 and BEAS-2B lung cells at concentrations ranging from 0.1 to 100 µg/mL CSC. As observed in **Fig.** **5**, as the CSC concentration increases, the viability of both cell lines decreases. However, we observed that between 0.1 to 10 µg/mL CSC did not significantly change the viability of these cells. In addition, this CSC concentration is in the range of physiological concentration that has been found in smoker subjects [28], [29], [30], [31]. Thus, in the subsequent experiments we used these CSC concentrations. When A549 and BEAS-2B serum-starved cells were transfected with E6/E7 and exposed to 0.1 and 10 µg/mL of CSC for 24, 48 or 72 h, a significant increase of viability was observed in comparison to non-exposed or non-transfected cells (**Fig.** **6**). These differences were statistically significant (p<0.001 or p<0.01). For A549 cells the maximum viability increase occurred when the cells were exposed to 0.1 µg/mL CSC (**Fig.** **6C**); for BEAS-2B this increase occurred at 10 µg/mL CSC (**Fig.** **6B**). Because the MTS-based proliferation method that we used is able to measure metabolic activity of viable cells, we confirmed these data using FACS. As shown in **Fig.** **7**, A549 cells showed a significant increase in proliferation rate when transfected with pLXSN E6E7 and exposed to 0.1 µg/mL of CSC with respect to those cells transfected with the empty vector and exposed to CSC (p<0.0001).

### Lung cells transfected with PLXSNE6E7 and acutely exposed to CSC increase significantly anchorage-independent growth

**Figure 8:**
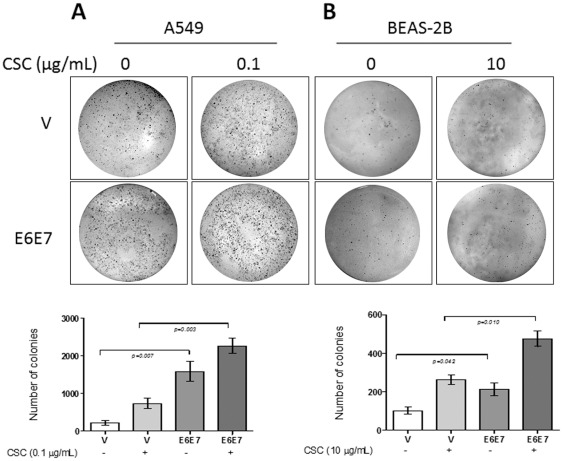
HPV-16 E6 and E7 expression in A549 (A) and BEAS-2B (B) cells exposed to CSC promotes an increased anchorage-independent growth. Below: Number of colonies per plate in A549 (left) and BEAS-2B (right) (mean ± SD, unpaired two-tailed Student's t-test. Each plate is representative of three independent experiments).

The tumoral A549 cells formed colonies in soft agar after transfection with pLXSN empty vector; however these colonies significantly increased in number in the presence of E6 and E7. Moreover, when A549 cells were exposed to 0.1 µg/mL CSC, the number of colonies was increased in mock and E6/E7 transfected cells, although this increase was significantly greater in E6/E7 transfected cells (p<0.01, **Fig.** **8A**). In addition, BEAS-2B developed a low number of colonies in the presence of empty pLXSN vector. In the presence of E6/E7 the cells showed an increased ability to grow in soft agar. When these E6/E7 transfected BEAS-2B cells were exposed to 10 µg/ml CSC, they showed an increased ability of anchorage-independent growth (p<0.05). However, BEAS-2B showed a less evident anoikis loss in the presence of E6/E7 and CSC compared to the tumoral A549 cells. Taken together, the A549 and BEAS-2B cells showed more anchorage independent growth in the presence of E6/E7 and when they were acutely exposed to CSC.

## Discussion

Previously we reported that HPV is present in subsets of lung carcinomas from some Latin American and Asian Countries. In addition, HPV was frequently integrated into the host genome with a low viral load [13], [32], [33]. Thus it seems plausible that this viral load requires other factors for carcinogenesis. Even though HPV has been detected in non-smoking and smoking subjects, the role of HPV in tobacco smoke associated carcinogenesis is frankly unknown [13]. An additional difficulty is the definition of the condition “smoker” or “non-smoker”; frequently this definition is based on clinical information submitted by the patients, so potential environmental exposition to tobacco smoke is possibly underestimated. Thus in this study we analyzed the possibility of a functional cooperation between HPV and tobacco smoke in lung epithelial cells. We transfected lung epithelial cells with constructs stably expressing HPV16 E6 and E7 oncoproteins that were subsequently exposed to CSC. Our findings showed that expression of high-risk E6 and E7 oncoproteins is associated with changes in the expression level and stability of important genes such as p53, involved in cell cycle regulation (**Figs.** **1** **and** **2**). In addition, hTERT and p16^INK4a^ expressions were strongly induced in non-tumoral lung cells (**Fig.** **3**). Previously it was reported that the high-risk E6 oncoprotein is able to cooperate with c-myc to induce telomerase expression in primary human keratinocytes [34]. Moreover, telomerase overexpression was demonstrated to occur in lung carcinomas associated with E6 expression [20]. On the other hand, p16^INK4a^ is a cyclin-dependent kinase inhibitor protein currently up-regulated in HPV-associated malignancies. We found that in BEAS-2B cells mRNA of this protein increased significantly its levels after transfection with the E6/E7 construct. Taken together, here we confirmed that E6 and E7 were functional in lung cells, inducing p53 alterations at the protein level with telomerase and p16IN4a overexpression.

When HPV16 E6/E7 transfected cells were exposed to CSC, the proliferative rate and anchorage-independent growth were significantly increased. In this respect, early studies carried out by Li et al. demonstrated that HPV was able to transform oral keratinocytes exposed to benzopyrene [35]. In addition, Park et al., reported that high-risk HPV immortalized oral cells may convert to a tumorigenic phenotype by chronic exposure to benzopyrene [36] or tobacco-related carcinogens [37]. Later it was reported that both benzopyrene and HPV infection contribute to the acquisition of an anti-apoptotic phenotype in oral cells [38]. Recently it was found that exposure of epithelial aerodigestive cells to CSC, activated the NF-κB pathway [39].

In our study, the analysis of proliferation rate, anchorage-independent loss and FACS suggest an additive effect between HPV16 E6/E7 oncoproteins and CSC for lung tumorigenesis. Interestingly, as commented before, tobacco smoke induces p53 accumulation because of its genotoxic effect on epithelial lung cells. However, high-risk E6 induces p53 degradation and thus prevents apoptosis of high-risk HPV infected cells. Taken together, accumulation of genotoxic damage in cells exposed to tobacco smoke is probably more intensive in HPV16 E6/E7 expressing cells. The translational implications of these findings remain to be elucidated.

Even though the International Agency for Research on Cancer (IARC) identified tobacco smoke a as carcinogen to humans [40], the mechanism for tobacco smoke-mediated carcinogenesis remains unclear. It is known that smoking is associated with molecular alterations related to stimulation of cell proliferation, thus multiple stages and pathways are involved. More than 4000 compounds have been identified in tobacco smoke; more than sixty of them with demonstrated carcinogenicity in humans are involved in initiation, promotion or progression of tumors [21]. Some tobacco compounds cause adducts in the DNA after activation by cytochrome P450 enzymes to form electrophilic molecules. The adduct formation may be related to TP53 or K-Ras mutations, as observed in patients with lung cancer [41]. In addition to DNA alterations, some components of tobacco such as nicotine are able to stimulate mitogenic responses and promote cell proliferation by binding to specific receptors such as the epidermal growth factor receptor (EGFR) and the nicotinic acetylcholine receptor (nAChR). Because the expression of these receptors is tissue-dependent, the response to cigarette smoke components such as nicotine shows tissue specificity [42]. The EGFR, whose expression is altered in malignancies, activates both the phosphatidylinositol-3 kinase (PI3K) and mitogen-activated/extracellular signal-regulated kinase (MAPK) pathways, which can potentially modulate activation of nuclear factors κB (NF-κB) and AP-1, respectively. On the other hand, it has been found that nAChR, an ion channel located in plasmatic membranes, is widely distributed in epithelial cells, keratinocytes, endothelial and non-small cell lung carcinomas (NSCLCs) [43], explaining the widely distributed tobacco smoke-associated diseases, including cancer [42]. It has been reported that both nicotine and 4-(Methylnitrosamino)-1-(3-pyridyl)-1-butanone (NNK) are able to induce lung cancer by binding to nAChR and activating PKC, Raf1, AKT, ERK1/2, and transcription factors such as Jun, Fos, and c-Myc [21], [42], [43], [44]. In addition, nicotine, nitrosamines and PAH can activate beta-adrenergic receptors (β-AR) and the aril-hydrocarbon receptor (AhR). The first of these is activated by catecholamines and NNK. AhR is a ubiquitous protein involved in regulating expression of genes associated with activation and detoxification of carcinogens [45]. In this respect, using microarrays it has been reported that high-risk HPV E6 and E7 oncoproteins are able to deregulate the AhR pathway in SCLC, a tumor strongly associated with the smoking habit [46].

Even though in this study a significant increase in proliferative rate and anchorage-independent growth was found, the mere expression of E6 and E7 oncoproteins was sufficient for alterations in the analyzed properties of lung cells. In consequence, is not possible to discard the possibility that E6 and E7 oncoproteins act independently of tobacco smoke. In fact, Ciotti et al using proteomic analysis reported that E6 and E7 oncogenes when overexpressed in A549 lung cells are able to upregulate the levels of Annexin III, gp96, transaldolase 1, between others [47]. In our study, a dramatic increase of proliferation and anchorage-independent growth was observed only after E6/E7 transfections (and hTERT overexpression), and acute cigarette smoke exposition incremented these properties.

A very important issue is if the CSC concentrations that were used in this study represent a real physiological exposure to tobacco smoke. The concentration of CSC is 40 mg/mL which corresponds to 6.4% of nicotine. In this report, lung cells were exposed to 0.1 and 10 µg/mL CSC which corresponds to 0.064 and 0.64 µg/mL nicotine. It has been reported that nicotine concentration ranged between 0.6 to 1.0 µg/mL into the tissue of smokers and 0.04 to 0.072 µg/mL in serum [28], [29]. Thus, the CSC concentrations used in this study are compatible with real physiological conditions.

In conclusion, in this study a functional cooperation between HPV16 E6/E7 oncoproteins and CSC, leading to an increase of proliferation rate and anchorage-independent growth of lung cells was shown. According to our knowledge, this is the first report addressing a potential cooperation between HPV and tobacco smoke in lung epithelial cells. Considering that HPV16 E6 and E7 oncoproteins are detected in a subset of lung carcinomas from subjects around the world who smoke or are exposed to tobacco smoke, they appear to be able to modulate the proliferative and tumoral behavior of cells exposed to tobacco smoke in bronchial epithelia. So it is plausible that in smoking subjects exposed to HPV infection, a functional interaction increases the possibility of carcinogenic progression. Additional functional and epidemiological studies are warranted to resolve this question.

## Materials and Methods

### Cell culture and transfections

Human cell lines A549 (lung adenocarcinoma, CCL-185), BEAS-2B (normal bronchial tissue, CRL-9609) and SiHa (HPV16 positive cervical carcinoma, HTB-35) were directly obtained from the American Type Culture Collection (ATCC) (Manassas, VA). These cell lines were maintained in RPMI-1640 (Invitrogen) supplemented with 10% inactivated fetal bovine serum (FBS) (Hyclone), 0.1 μg/mL gentamicin (Invitrogen), 1 U/mL penicillin and 1 μg/mL streptomycin (Invitrogen) and incubated at 37°C in a 5% CO_2_ atmosphere incubator. The cells were checked for mycoplasma infection using standardized protocols. A549 and BEAS-2B cells were transfected with the retroviral vectors pLXSN and pLXSN containing the HPV-16 E6/E7 ORFs (pLXSNE6, pLXSNE7 and pLXSNE6E7) (kindly provided by Dr. Massimo Tommasino; IARC, Lyon, France). Transfection assays were made using lipofectamine 2000 (Invitrogen) according to the manufacturer's instructions. Briefly, one day before transfection cells were seeded in six-well plates with 3 mL of basal RPMI-1640 medium without antibiotics. For each plate, 4 μg of pLXSN recombinant plasmids were mixed with 250 μL of RPMI-1640 basal medium without antibiotics. The mixture was then combined with 10 μL of lipofectamine 2000 (Invitrogen) in 250 μL of RPMI-1640 and incubated for 20 min at room temperature. The plasmid mixture was directly added to the cells containing RPMI-1640 supplemented with 5% FBS without antibiotics. The cells were incubated for 18 h at 37°C and then the cell culture medium was discarded; the cells were washed with phosphate buffered saline pH 7.4 (PBS) and incubated with RPMI-1640 supplemented with 5% FBS and 0.5 mg/mL G418 for selection. After 2 to 3 weeks, the resulting colonies were pooled and used for subsequent analysis.

### RNA purification, cDNA preparation and reverse-transcriptase polymerase chain reaction (PCR)

Cellular RNA was isolated using 1 mL of Trizol reagent (Invitrogen) according to the manufacturer's instructions. The RNA concentration and purity was determined using a Nanodrop1000 spectrophotometer (Thermo Scientific) and 2 μg of RNA was treated with RQ1 DNAse (Promega) at 37°C for 1 h and denatured at 65°C. cDNA was synthesized in a 20 μL reaction volume containing DNAse-treated RNA (2 μg), 1 U RNAse inhibitor (Promega), 0.04 μg/μL random primers (Promega), 2 mM dNTP (Promega) and 10 U Moloney murine leukemia virus (MMLV) reverse transcriptase (Promega). The reaction mixture was incubated for 1 h at 37°C followed by incubation at 65°C for 10 min. The cDNA samples were subjected to PCR amplification with specific primers for HPV16 E6/E7, p53 and GAPDH (**Table** **1**). For GAPDH cDNA amplification, 1 μL of the prepared cDNA was added to a tube containing 1X reaction buffer; 2.5 mM MgCl2; 0.2 mM dNTP; 0.52 μM primer pairs, and 0.08 U GoTaq Flexi DNA polymerase (Promega). PCR amplification was performed under the following conditions: denaturation at 94°C for 5 min followed by 35 cycles of 94°C for 30 s, 55°C for 30 s, 72°C for 30 s and final extension for 5 min at 72°C. For E6, E7 and p53 cDNA amplification, we used 1 μL of cDNA, 1X GoTaq Flexi buffer; 0.2 mM dNTP; 2.5 mM MgCl2; 0.5 μM primers and 0.08U GoTaq Flexi DNA polymerase (Promega). PCR amplification was performed under the following conditions: denaturation at 95°C for 5 min, followed by 35 cycles at 95°C for 1 min, 55°C for 1 min, 72°C for 1 min and final extension for 10 min at 72°C. Amplified products were characterized using 3% agarose gel electrophoresis, stained with ethidium bromide and observed with a UV-transilluminator (Vilver Lourmat).

**Table 1:** Primers used in this study.

Gene	Primer	sequence	Size product (bp)
GAPDH	Sense	CGGGAAGCTTGTCATCAATGG	
	Antisense	CATGGTTCACACCCATGACG	221
E6	Sense	CAACAAACCGTTGTGTGAT	
	Antisense	CGTGTTCTTGATGATCTGC	162
E7	Sense	ATG CATGGAGATACACCTAC	
	Antisense	CATTAACAGGTCTTCCAAAG	252
p53	Sense	GGAGCCGCAGTCAGATCCTA	
	Antisense	GGGGACAGAACGTTGTTTTC	96
p16^INK4a^	Sense	TCGGGTCGAGGAAGGTGA	
	Antisense	TCATGATGATGAGGGCAGCG	102
hTERT	Sense	GCGGAAGACAGTGGTGAACT	
	Antisense	ACCTGGAGTAGTCGCTCTGC	150

### Quantitative real-time PCR (qRT-PCR)

The primers used for E6, E7, hTERT and p16^INK4a^ amplification were described in Table 1. For hTERT, the reaction mixture was prepared as follows: 0.4 µM primer F, 0.4 µM primer R, 1.5 mM MgCl2, 1X SensiMix (Bioline, Taunton, Massachusetts, USA) and 1 µL cDNA. PCR amplification was performed as follows: 10 min incubation at 95°C; 45 cycles of 15 s at 95°C, 20 s at 55°C and 20 s at 72°C. For p16, E6, E7 and GAPDH the reaction mixture was prepared as follow: 0.5 µM primer F, 0.5 µM primer R, 1.5 mM MgCl2, 1X SensiMix and 1 µL cDNA. For p16^INK4a^, the PCR amplification was performed as follows: 10 min incubation at 95°C; 45 cycles of 10 s at 95°C, 10 s at 55°C and 10 s at 72°C. For E6 and E7 the amplification conditions were the same that were used for hTERT. GAPDH amplification was used for data normalization. The PCR amplification was performed as follows: 10 min incubation at 95°C; 45 cycles of 15 s at 95°C, 20 s at 55°C and 20 s at 72°C. The PCR reactions were carried out using Rotor Gene 6000 (Corbett Research) real-time PCR equipment. The specificity of amplification was confirmed using dissociation analysis starting at 60°C.

### Protein purification and Western Blotting (WB)

Total protein was extracted from cells with a lysis buffer (100 mmol/L Tris pH 8.0, 1% SDS) containing a protease inhibitor cocktail (Sigma-Aldrich). The cells were incubated at 4°C for 1 h, sonicated on ice for 20 s and centrifuged at 12,000 G for 10 min. The protein concentration was determined using Pierce BCA protein Assay Reagent kit (Thermo). For p53 blots; 25 µg of the extract was loaded per well on 10% polyacrylamide gels. Following SDS-PAGE, proteins were transferred onto a nitrocellulose membrane in a Trans-Blot semi-dry electrophoretic transfer cell (BioRad) (20 min, 15 V). The membranes were blocked in 5% dry milk–TBS–Tween 20 and incubated for 16 h at 4°C with a primary monoclonal anti-p53 antibody (DO7 clone) diluted 1∶1000 (BD Biosciences). After extensive washing with TBS-Tween 20, the membrane was developed using an ECL kit according to the manufacturer's instructions (Amersham Biosciences). β-actin (1/10.000) (Abcam) was probed in all samples to exclude false negative results and cell lysates from CasKi cells (containing HPV16) were used as positive controls.

### Smoke exposures and CSC preparation

The transfected A549 and BEAS-2B cell lines were maintained at 80–90% confluence with RPMI-1640 basal medium, serum-starved for 24 h previous to CSC (Murty Pharmaceutical, Lexington, KY, USA) exposition. The CSC dilutions in basal RPMI-1640 medium were freshly prepared for each experiment in a range from 10^−3^ to 50 µg/mL using a 40 mg/mL stock solution. Dimethyl-sulfoxide (DMSO) was used as negative control. The CSC stock solution was aliquoted and stored light-protected at −80°C.

### Cytotoxicity CSC assays

A549 and BEAS-2B transfected cells were trypsinized with an EDTA-trypsin mixture (Invitrogen) and counted using Trypan blue exclusion staining with a Neubauer chamber. Briefly, 5×10^3^ cells were prepared and incubated on 96-well plates with RPMI-1640 (Invitrogen, Grand Island, NY, USA) supplemented with 10% FBS (Hyclone, Logan, UT, USA), penicillin/streptomycin mixture and 0.1 µg/mL gentamicin (Invitrogen). The CSC was added to the cells and incubated at 37°C in a 5% C0_2_ atmosphere incubator. After 72 h of incubation, thirty µL of MTS reagent (Promega) were added to each well and the plate was incubated for three h at 37°C; the absorbance was measured at 492 nm using a microplate reader (model Anthos 2001).

### Viability and proliferation assays

Transfected cell lines were grown to 80–90% confluence and incubated with serum-starved RPMI-1640 medium in 60 mm plates for 24 h. The cells were trypsinized with EDTA-trypsin mixture (Invitrogen) and counted using Trypan blue exclusion staining with a Neubauer chamber. 5×10^3^ cells were cultured in a 96-well flat-bottomed microtiter plate with RPMI-1640 basal medium and alternately supplemented with 0.1 and 10 µg/ml of CSC for periods ranging from 24 to 120 h, using DMSO as control. Finally, thirty µL of [3-(4,5-dimethylthiazol-2-yl)-5-(3-carboxymethoxyphenyl)-2-(4-sulfophenyl)-2H-tetrazolium, inner salt; MTS] (Promega) were added to each well, the plate was incubated for 3 h at 37°C and the absorbance was measured at 492 nm using a microplate reader (Anthos 2001).

### CFSE proliferation assays

A549 transfected and non-transfected cells were passaged at 80–90% confluence in 60 mm plates without serum for 24 h at 37°C in a 5% CO_2_ atmosphere incubator. Then the cells were trypsinized with EDTA-trypsin 1X (Invitrogen) and counted using trypan blue staining. 1×10^6^ cells were treated with 0.5 µM carboxifluorescein succinimidyl ester (CFSE) for 10 min at 37°C. Then 5×10^4^ cells were deposited in 6-well plates in RPMI-1640 basal medium with 10% FCS and exposed to 0.01 and 10 µg/mL CSC during 48 h. The cells were washed with 1X PBS, suspended with 1X trypsin-EDTA and analyzed by flow cytometry in the FACS Cantoll equipment. (Beckett Dickinson, Mississauga, Ontario, USA), considering a universe of 10,000 cells.

### Anchorage-independent growth assays

The transfected cells exposed to 0.01 and 10 µg/mL of CSC for 72 h were trypsinized using 1X Trypsin-EDTA mixture and counted using trypan blue staining. Thus 1×10^4^ cells were suspended in 0.33% Bacto-agar dissolved in a mixture containing 2X RPMI-1640 basal medium, 12.5% FBS, 1X RPMI-1640 supplemented with 0.01 or 10 µg/mL CSC. The cells were cultured in six-well plates containing 0.5% agar for 1 month. The cells were fed twice each week with 0.5 mL of RPMI-1640-10% FBS supplemented with 0.01 or 10 µg/mL CSC. Finally, the cells were stained using 0.005% crystal violet for 1 h and photographed.

### Statistical analysis

Overall numeric data were analyzed considering averages and standard deviations. The comparison between CSC treated groups and controls were made considering a non-paired statistical analysis with 95% confidence interval using Prism 4.0 software (GraphPad Software, San Diego, CA, USA). A p value less than 0.05 was considered as statistically significant.
